# Critical health literacy: reflection and action for health

**DOI:** 10.1093/heapro/daac114

**Published:** 2022-09-01

**Authors:** Thomas Abel, Richard Benkert

**Affiliations:** Institute of Social and Preventive Medicine University of Bern, Bern, Switzerland; Institute of Social and Preventive Medicine University of Bern, Bern, Switzerland

**Keywords:** health inequality, empowerment, social theory

## Abstract

Health literacy research is growing rapidly and broadly; however, conceptual advances in critical health literacy (CHL) seem hampered by a lack of a clear definition. In this paper, we refer to key features of the concept as identified in earlier works, offer a new definition of CHL and briefly discuss its theoretical roots. Reflection and action are suggested as the two constituent components of CHL. Consequences for future research are also discussed.

## BACKGROUND

In public health and health promotion, health literacy is considered an important resource for individual and collective empowerment (Nutbeam, 2000; Kickbusch, 2001). Among the different types of health literacies, critical health literacy (CHL) has recently gained increased attention in health research with the number of publications substantially increasing since 2010 (Benkert and Abel, 2022). It was originally drawn from the concept of critical literacy, which focused on individuals’ abilities to analyse and use information as a means of greater autonomy and empowerment (Nutbeam, 2000; Sykes et al., 2013). Beyond this, CHL was also introduced to address socio-political dimensions of health literacy (Sykes et al., 2013). These socio-politico dimensions refer to agency as the ability to bring about social and political change and—through an improved capacity to act on the social determinants of health (SDOH)—overcome ‘structural barriers to health’ (Nutbeam, 2000, p. 267). Thus, CHL is not limited to achieving individual health benefits; it also entails empowering communities and reducing social health inequalities.

Although Nutbeam’s foundational work introduced the concept of CHL, he did so without providing a concise definition. In principle, this openness could have meant opportunities to develop definitions of CHL that address its distinct features and account for dynamic, evolving contexts. However, without such attempts and a concise definition, later works addressing CHL often fell back onto notions of ‘critical literacy’ losing socio-political dimensions originally considered a key component of CHL (Sykes et al., 2013). Over time, as Sykes and colleagues (2013) pointed out, critical literacy has almost become the *de facto* definition of CHL. Yet, although the definition of critical literacy alludes to greater individual control over life events, consequent notions of CHL tend to ‘lack... specific reference to social and political action and existence at a population level’ (Sykes et al., 2013, p. 8). Therefore, ‘these elements are in danger of becoming lost so distorting the original meaning and emphasis’ (Sykes et al., 2013, p. 8). Although there are many descriptions of what CHL entails, to the best of our knowledge to date, there is still no clear definition of this term that accounts for its conceptual origins regarding empowerment and social inequality.

Current CHL approaches address an awareness of social and cultural conditions as prerequisite to acquire competencies required to act for reducing health inequalities (Chinn, 2011); however, these approaches, and health literacy research in general, including research on CHL, often omit theoretical support (Pinheiro, 2021). Referring to CHL’s theoretical underpinnings, Pithara (2020) notes that ‘CHL adopts an emancipatory, empowerment-led understanding, where people are cognizant of social, economic and environmental determinants of health and are able to tackle these through community action’ (p. 2). Intervention frameworks have been designed to promote awareness, understanding and reflection by educating individuals and communities about SDOH; thereby contextualizing CHL and teaching community members how to empower people to ameliorate health inequalities (Mogford et al., 2011). Yet, until today, CHL’s basic role in the social reproduction of health inequalities remains an open issue. With socio-political features of CHL as a core attribute (Nutbeam, 2000; Sykes et al., 2013), it seems warranted that social theories would guide CHL research and practice; still, theoretical frames in CHL research are markedly absent. One noticeable exception is the work of Sykes and Wills (2018, 2019). These authors suggest a socio-critical approach, tracing the concept of CHL back to the idea of ‘critical consciousness’ developed by Brazilian educator and philosopher Paulo Freire.

In what follows, we present our definition of CHL and briefly revisit Freire’s *Pedagogy of the Oppressed* (1970/2005) as it provides theoretical support for CHL’s socio-emancipatory function. We provide examples of how the new definition can illuminate current CHL assessments. We then refer to Pierre Bourdieu’s ‘theory of practice,’ as an example of a theoretically meaningful application of our new definition of CHL within the broader health inequality discourse. Our definition of CHL is meant as a starting point for further discussion, development, and application in future research about CHL and social health inequality.

## DEFINING CRITICAL HEALTH LITERACY

Building upon those earlier works referenced above, we focus on reflection and action as two constituent components for our definition of CHL for public health and health promotion. We define CHL as *the ability to reflect upon health determining factors and processes and to apply the results of the reflection into individual or collective actions for health in any given context*. Developed with a focus on empowerment and health inequalities, our definition is suitable and flexible to serve a wide range of research questions and address more specific forms of CHL that can consider the social contexts in which CHL operates. For both purposes, reflection and action remain the two major constituent components of CHL, which allow addressing individual and collective agency and its structural conditions.

## ROOTS OF REFLECTION AND ACTION FROM PAULO FREIRE

Providing useful theoretical grounding for conceptualizing CHL, health literacy scholars have drawn from Freire’s (1970/2005) critical pedagogy work (Gould et al., 2010; Chinn, 2011; Mogford et al., 2011; Estacio, 2013; Sykes et al., 2013; Renwick 2017; Sykes and Wills, 2018). We argue the social conditions of health find strong anchoring among Freire’s components: reflection and action. Freire explores how oppression is socially installed and how to liberate from it. He challenges traditional approaches in education, which he calls the ‘education as the practice of domination’ in a hierarchically structured society (Freire, 1970/2005, p. 81). According to the so-called ‘banking concept’ of education, students are treated as recipients of knowledge or ‘depositories’, deprived of the possibility to acquire ‘critical consciousness’ (Freire, 1970/2005, pp. 72, 73, 83) and thus depleted of the ability to liberate themselves from dominating systems.

For Freire, empowerment in general presupposes that social determinants are questioned. He advocates for a ‘problem-posing education’ pedagogical theory in which education is understood as ‘the practice of freedom’ and where students are treated as people who are educated into being ‘critical thinkers’ in a participatory and dialogical way of learning (Freire, 1970/2005, p. 81). He explains, ‘In problem-posing education, people develop their power to perceive critically the way they exist in the world with which and in which they find themselves’ (Freire, 1970/2005, p. 83). In other words, all individuals need to acquire a critical attitude towards the socio-cultural reality that shapes their lives—*reflection*. Furthermore, individuals must also develop the ability to change their life-world through *action*. With reference to Prilleltensky (1989, p. 800), Jemal (2017) notes that ‘the process whereby people achieve an illuminating awareness both of the socio-economic and cultural circumstances that shape their lives and their capacity to transform that reality is parallel with an empowerment process’ (p. 3). A person’s capability to reflect upon their world and the action it takes to change it are considered central instruments for empowerment (Freire, 1970/2005, p. 79).

Reflection and action have been conceptualized as the two main components of Freire’s critical consciousness. Reflection means ‘examining everyday realities to analyse relationships between personal contexts and the wider social forces of structural oppression (e.g. social, economic and political environments) that restrict access to opportunity and resources, and thus, sustain inequity and perpetuate injustice that limit well-being and human agency’ (Jemal, 2017, p. 6). Action refers to ‘the overt engagement in individual or collective action taken to produce socio-political change of the unjust aspects (e.g., institutional policies and practices) of society that cause unhealthy conditions’ (Jemal, 2017, p. 6). Others describe critical action as an ‘individual’s objective ability or potency to act given structural constraints’ (Campbell and MacPhail, 2002, p. 333). In fact, the ability of individuals or communities to reflect and act with the aim of improving structural conditions for health was already addressed in Nutbeam’s (2000) first delineation of CHL. He puts it like this: CHL ‘reflects the cognitive and skills development outcomes which are oriented towards supporting effective social and political action, as well as individual action’ (p. 265). In a further remark, he states, implying reference to reflection, that CHL encompasses the ‘development of skills which investigate the political feasibility and organizational possibility of various forms of action to address social, economic and environmental determinants of health’ (Nutbeam, 2000, p. 265). In this regard, it has been pointed out that CHL is linked to understanding the determinants and policy context of health, as well as of opportunities to challenge these determinants and policies. At the same time, it is linked to motivation and actual action at political and broader social levels (Sykes et al., 2013, p. 5).

## CONSEQUENCES FOR CRITICAL HEALTH LITERACY ASSESSMENTS

Several studies have provided empirical measures assessing selected aspects of CHL (e.g. Ishikawa et al., 2008; Osborne et al., 2013; Abel et al., 2015). Most of these, however, were focused on assessing individuals’ abilities to critically evaluate information or their critical literacy. From a theoretical perspective, empirical measures of empowerment linked to reflection and action that could address social forces at structural and individual levels in comprehensive, theoretically coherent ways are missing.

While reflection and action have rarely been measured explicitly in CHL research to date, some empirical instruments are available, addressing single elements of empowerment and political components of CHL, such as Chinn and McCarthy’s (2013) All Aspects of Health Literacy Scale (AAHLS); Matsumoto and Nakayama’s (2017) Health Literacy on the Social Determinants of Health Questionnaire (HL-SDHQ); and Shannon and Parker’s (2020) Health Communication Questionnaire. In this respect, our definition of CHL might prove useful to illuminate previous findings. For example, Matsumoto and Nakayama (2017) addressed socio-political dimensions of CHL and the ability to bring about health-relevant changes at the population level (i.e. community empowerment). Their HL-SDHQ tool points to a more advanced assessment of CHL since it includes items about an individual’s ‘ability to cooperate in the creation of a fair society in which everyone can live a healthy life’ (p. 6) or to ‘involve oneself in politics and public administration on various health-related issues’ (p. 7). Applying our new definition of CHL can facilitate a focused interpretation of their findings. For instance, those two items clearly addressing the ‘action’ part of CHL, while also encouraging researchers to pursue theory-guided assessments.

Another example of how the new definition of CHL can illuminate previous findings comes from Chinn and McCarthy’s (2013) AAHLS instrument. This measure contains questions that indirectly provide information about reflection and action. For instance, one question elicits the ‘perceived possibilities’ of the individual’s contribution to community health, alluding to ‘reflection.’ Another question from the AAHLS deals with active participation in health-promoting processes, implying the action part of CHL. Reflection and action and their acquisition and application are dependent on the context in which people strive for better health (Abel, 2008a; Nutbeam, 2009; Pithara, 2020; Pinheiro, 2021). Recent studies have used qualitative research methods for deeper understandings of inequality dynamics in the context of health care, including the application of CHL (de Wit et al., 2017; Dubbin et al., 2021). Insights obtained via qualitative methods and analyses may also provide evidence for interventions for improving CHL, spurring community empowerment and reducing health inequalities (Gould et al., 2010; Mogford et al., 2011).

Since any definition of CHL for health research should facilitate empirical study by providing theoretical guidance without restricting the breadth of immanent research questions, our definition above is kept sufficiently broad to support empirical measures for different health themes and fields (e.g. health-relevant lifestyles and consumer markets, healthcare contexts, workplace, and housing conditions). Moreover, previous empirical approaches to health literacy have been subject to basic criticism for measures that tend to address health issues from class-biased or context-insensitive perspectives (Abel, 2008a; Pinheiro, 2021), such as those addressing health lifestyle items that are out of reach or less relevant for health in materially deprived living conditions. For example, measures assessing individuals’ knowledge about selecting healthy, yet often more expensive foods or risks overestimating agency, while downplaying the role of structural constraints for action and reflection. Thus, definitions of CHL that aim at supporting empirical study should facilitate a broad range of research questions about inequality, while also avoiding conceptual biases.

## CRITICAL HEALTH LITERACY AND THE REPRODUCTION OF INEQUALITY—PIERRE BOURDIEU

Considering our definition of CHL, questions about social inequalities and inequities arise: how does CHL affect health, health behaviours and their social conditions? What are the individual and communal conditions in which CHL can best be acquired? What are the social, economic and cultural resources needed for a successful application of CHL in various contexts, such as healthcare systems, workplaces and leisure time activities? Such questions refer directly to unequal and inequitable chances for an individual’s agency to achieve good health. They also lead to basic questions about structural factors and processes, including broader questions about how CHL ties into the dynamics of reproducing social inequalities at the population level.

Theoretical guidance seems warranted to address these larger questions and anchor the concept of CHL firmly within social health inequality discourse. To assist more comprehensive understandings of links between a person’s structural living conditions and their chances to reflect upon and act on SDOH (i.e. their CHL), we call upon Pierre Bourdieu’s theory of practice (Bourdieu, 1977a; Bourdieu and Passeron, 1990) and his concepts of habitus and capital (Abel and Frohlich, 2012). Bourdieu’s theory of practice has been successfully applied to study how health inequalities are reproduced (Williams, 1995; Veenstra 2007; Schori et al. 2014; Jeong and Veenstra, 2017), including social patterning of health lifestyles, beliefs and health behaviours (Cockerham et al., 1997; De Clercq et al., 2017; Gagné et al., 2018; Kandt, 2018).

The concepts of habitus and capital bear specific relevance for theoretically grounding CHL. In a Bourdieusian approach, reflection and action are primarily of social origin. They can be explained as an expression of the habitus that ‘serves as a cognitive map or set of perceptions that routinely guides and evaluates a person’s choices and options’ (Cockerham, 2005, p. 61). The habitus ties an individual’s reflection to action and operates as a constant, yet dynamic and flexible filter and guide across different contexts. Researchers of health inequality have used this concept to explain the reproduction of inequalities via health-relevant social practices and lifestyles (Cockerham, 2005; Veenstra, 2018). Habitus and health lifestyles are closely tied to the availability of economic, social and cultural capital; therefore, they are inextricably linked to an individual’s social position in a ‘field of struggles’ (Bourdieu and Wacquant, 1992, p. 101), such as those over resources needed to live healthy lives or achieve good health across different contexts (Khawaja and Mowafi, 2006; Missinne et al., 2014; Deshmukh et al., 2015; Paccoud et al., 2020).

Among the different forms of capital, cultural capital bears special importance for health literacy research (Abel, 2007; Adkins and Corus, 2009). Cultural capital allows an individual to succeed in the competition over privilege, power, and status (Bourdieu and Passeron, 1990; Bourdieu, 1977b). Applied to health, the concept of cultural capital addresses issues of unequal distribution of health and its resources in a theoretically coherent way (Abel, 2008b; Shim, 2010; Veenstra and Abel, 2019). Cultural capital comes in three different types, namely, institutionalized, objectivized and embodied cultural capital. Educational degrees and other formal qualifications, typically issued by accredited institutions of higher education, are the most widely used measures of *institutionalized cultural capital*. Material forms, objects or goods of cultural value signifying higher or superior social status (e.g. art in the home, possession of highbrow books or clothes) indicate an individual’s *objectivized cultural capital*. Finally, e*mbodied cultural capital* refers to various kinds of behaviours, skills and socially relevant, valued knowledge acquired through formal and informal education in schools, families, sport clubs, etc. (Abel, 2007, 2008b; Oude Groeniger et al., 2020). It comprises health-relevant tastes, preferences and dispositions that are physically embodied, part of the habitus and practised through lifestyles (Cockerham, 2005). Health literacy broadly defined can be understood as part of this embodied cultural capital, and with it, CHL addresses social conditions and processes operating in the reproduction of health inequalities (Abel, 2007). Explaining CHL as part of an individual’s cultural capital, health-related habitus and lifestyles— with the latter structured along the lines of social class, status or milieu—thus provides a theoretically coherent way of linking CHL to the prevailing social structures of health inequalities.

Moreover, a theoretical approach via cultural capital identifies two interrelated functions of CHL. First, being part of an individual’s cultural capital, CHL can strengthen individual and collective agency, operating as a *resource for individuals* in their pursuit of better health. Second, since chances to acquire and use CHL are unequally distributed along the lines of established social hierarchies (e.g. via educational systems), it operates as a *transmitter of collective inequality* in different contexts. For example, contexts where there is group-based competition over health resources and power, community struggle over environmental resources (King et al., 2021), or patients’ struggles over access to quality health services (Shim, 2010; Rasmussen et al., 2021).

## DISCUSSION

From its beginning, CHL has been discussed as a concept that illuminates the factors and processes at work when individuals actively deal with health matters by critically considering the social conditions of health. However, in the absence of a clear definition of CHL most research approaches showed a neglect of the concept’s focus on social conditions and socio-political features (Sykes et al., 2013; Chinn, 2011; Chinn and McCarthy, 2013; Guzys et al., 2015; Diviani, 2019). The new definition we offer in this paper is geared towards two components of individual and collective agency: reflection and action on health matters. It considers SDOH as structural factors and recognizes contextual conditions for people to acquire and apply CHL.

Along with Freire and other critical researchers, we propose that reflection and action need to be linked conceptually and practically to reach empowerment for health (Rubinelli et al., 2009; Chinn, 2011; Mogford et al., 2011; Estacio, 2013; Sykes et al., 2013; Renwick, 2017; Sykes and Wills, 2018). This is because action without purposeful reflection runs the risk of mere ‘activism’ (Freire, 1970/2005, p. 88); and reflection without action might result in intellectual discovery only or what Freire identified as empty ‘verbalism’ (p. 87). CHL should capture the interplay of reflection and action, particularly in times when the contextual conditions for health become more and more complex. Many healthcare systems today increasingly turn into diversified markets with vested interest groups competing over profits and patients or consumers, making it progressively difficult for individuals and whole population groups to critically choose and act according to that choice (e.g. selecting healthcare services and insurance plans). CHL can and should address the SDOH but also the basic principles of power, whether economic markets or political systems.

We further suggested that Bourdieu’s theory of practice might be a viable option for anchoring CHL to the broader health inequality discourse. Again, our discussion is meant only as a starting point. Future studies might show how Bourdieu’s work can provide further and more specific contributions to situate CHL firmly in the context of social reproduction. However, other social theories can provide support for applying the new definition of CHL in health inequality research. Linking reflection and action to understand the basic components of agency and how it contributes to social reproduction or how it may lead to social change, opens CHL to insights from capabilities theory (Sen, 1985/1993; Robeyns, 2005; Abel and Frohlich, 2012; Pithara 2020) and health lifestyle theory (Cockerham, 2005). These theories both address fundamental issues of structure and agency in health inequalities. Future studies might explore questions about CHL’s role in individuals’ freedom to achieve health or the importance of CHL for the practice of health lifestyles to achieve better health and social status.

Our new definition also seeks to overcome some of the theoretical limitations previous approaches faced. Defined this way, CHL avoids *individual reductionism*—a point of critique for previous health literacy approaches (Guzys et al., 2015). By stressing reflection and action on social factors that determine health, as well as including the contextual conditions, individuals and communities strive for better health within, these can safeguard against risks of individualizing health literacy approaches. From our new definition, questions for future studies arise, such as how reflection can strengthen contextualizing health actions and strengthen individuals as agents and experts for health in their own life worlds. The focus on refection and action under conditions of social inequality suggests even farther-reaching topics. For instance, CHL theory and measurement might need to consider issues of intersectionality when addressing questions about what form of health inequities are at stake in specific contexts. Drawing on works from Crenshaw (1991) and those writing drawing from intersectionality theory (Collins, 2015) might also help advance the concept of CHL and augment critical discussions of health literacy in general.

The new definition also avoids too much *normativity* and the risks that come with it. As Huber et al. (2012) note:

the health literacy movement, at least in its current form, operates in a top-down model, where the establishment is primarily prescribing action plans designed to identify individuals with limited or low health literacy and provide interventions that seek to improve one’s ability to comprehend and use health information in appropriate ways. (p. 440)

Our definition of CHL points to a different direction, namely critical reflection by individuals opposing highly normative ‘prescriptions’ and instead, critically thinking about health, its social conditions, and the manifold interests of those involved in its production and distribution. When applied in research about SDOH, our new definition might be helpful to overcome the *prevailing divides* into ‘structural versus behavioural factors’ and ‘material versus non-material factors’ (Macintyre, 1997). Both divides are implicitly opposed by our definition that links structure and agency and stresses critical reflection and action relevant at all levels.

Although our discussion above has pointed to pending issues in theory development, the new definition of CHL also has implications for empirical research. Empirical studies on CHL can benefit from a theory-based definition in the development of new assessment tools in several ways. First, reflection and action define a clear focus for developing new indicators (quantitative approaches) and for observing systematically (qualitative approaches). For example, the new definition does not include value-laden criteria, for example, for what is a ‘high or low’ CHL. This will allow and ideally encourage future studies to develop measures of CHL that account for contextual variation in the meaning and effectiveness of reflection and action. For current studies on CHL exploring the importance of reflection and action for health (e.g. Haugen et al. 2022), the new definition may provide additional theoretical support for their empirical measures. The interplay between reflection and action will present a key challenge for future research then—ideally addressed through mixed-methods approaches. Second, a focus on the social conditions of health allows empirical research on CHL to integrate (i.e. draw from and feedback into) established research fields such as the ‘SDOH’, ‘social class, capital and health’ theory, or ‘health lifestyles’ research. Third, the basic proposition that CHL includes yet goes beyond the individual to account for collective patterns in the acquisition and application of CHL requires that data should be collected on each societal level, ranging from individuals to families to communities and even countries (e.g. EU Health Literacy Survey). Similarly, interventions should lay a focus on the social conditions that facilitate CHL at all relevant levels. Together, these three points indicate how applying our new definition of CHL can assist and complement future empirical studies in developing new measures and producing knowledge relevant for interventions that aim to strengthen individual and collective agency to reduce social health inequalities.

## CONCLUSIONS

Defining CHL based on reflection and action as its two constituent components can provide a focus for its application in research on health inequalities. Guidance from social theory can facilitate coherence in its definition and strengthen its application in empirical studies. Our definition and brief discussion may serve as a starting point for advancements needed to utilize and realize the full potential of CHL in future health promotion research and practice.
